# Vanishing bile duct syndrome induced by Psoraleae Fructus: A case report and literature review

**DOI:** 10.1097/MD.0000000000047483

**Published:** 2026-01-30

**Authors:** Min Liu, Chuang Lei, Hong-Ling Tian, Qing-Hai Wang, Xi-Yang Dong

**Affiliations:** a Department of Infectious Diseases, Changde Hospital, Xiangya School of Medicine, Central South University (The First People’s Hospital of Changde City), Changde, China.

**Keywords:** drug-induced liver injury, liver biopsy, Psoraleae Fructus, vanishing bile duct syndrome

## Abstract

**Rationale:**

Certain drugs can trigger inflammatory responses and necrosis in the interlobular bile duct epithelial cells of the liver. Persistent damage may cause bile duct disappearance and biliary cirrhosis. When over 50% of bile ducts are lost, drug-induced vanishing bile duct syndrome (VBDS), a rare yet severe clinical condition, can be diagnosed.

**Patient concerns:**

A 67-year-old woman had a radius fracture and took Chinese herbal medicine containing Psoraleae Fructus (PF) for 1 week, then developed jaundice and pruritus.

**Diagnoses:**

Laboratory tests confirmed cholestatic liver injury, imaging studies ruled out biliary obstruction, and liver biopsy showed progressive disappearance of interlobular bile ducts, consistent with a VBDS diagnosis.

**Interventions:**

The patient received plasma exchange and hepatoprotective therapy.

**Outcomes:**

After treatment, her liver function gradually improved.

**Lessons:**

PF may induce VBDS via immune or toxic mechanisms.

**Conclusion:**

Clinicians should maintain a high level of suspicion for PF-associated liver injury and enhance monitoring and risk assessment during its clinical use.

## 1. Introduction

Vanishing bile duct syndrome (VBDS) is a rare syndrome characterized by a reduction in intrahepatic bile ducts as the main pathological feature and cholestasis as the main clinical feature. It can be caused by various factors, including congenital anomalies, immune disorders, tumors, drugs, toxins, infections, graft-versus-host disease, and primary biliary cirrhosis.^[1,2]^ The incidence of VBDS is low, accounting for only 0.5% of small bile duct diseases. Among all VBDS, drug-induced vanishing bile duct syndrome (D-VBDS) accounts for 7%.^[3]^ Common drugs that cause VBDS include antibiotics, nonsteroidal anti-inflammatory drugs, antidiabetic drugs, lipid-lowering drugs, anticancer drugs, biological agents, and Chinese herbal medicines (traditional Chinese medicine), especially those utilized for managing bone pain.^[4,5]^ Psoraleae Fructus (PF) has been widely used in the prescription of tonifying kidney and strengthening bones. Although clinical studies have demonstrated that psoralen exposure is associated with dose-dependent hepatotoxicity,^[6]^ the development of VBDS as a sequela of psoralen-induced liver injury remains seldom reported in the medical literature. Here, we report a typical case of VBDS caused by PF.

## 2. Case report

**Figure 1.**
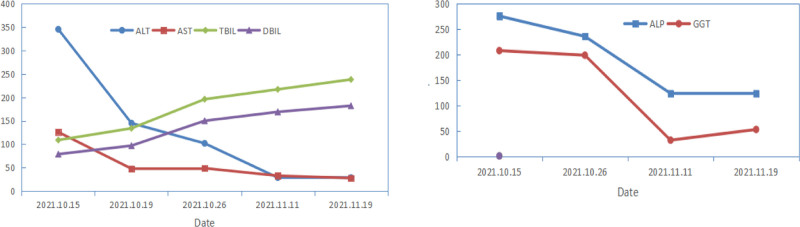
Trend of LFTs during prehospital. ALP = alkaline phosphatase, ALT= alanine aminotransferase, AST= aspartate aminotransferse, DBIL = direct bilirubin, GGT = gamma-glutamyl transferase, LFT = liver function test, TBIL = total bilirubin.

**Figure 2.**
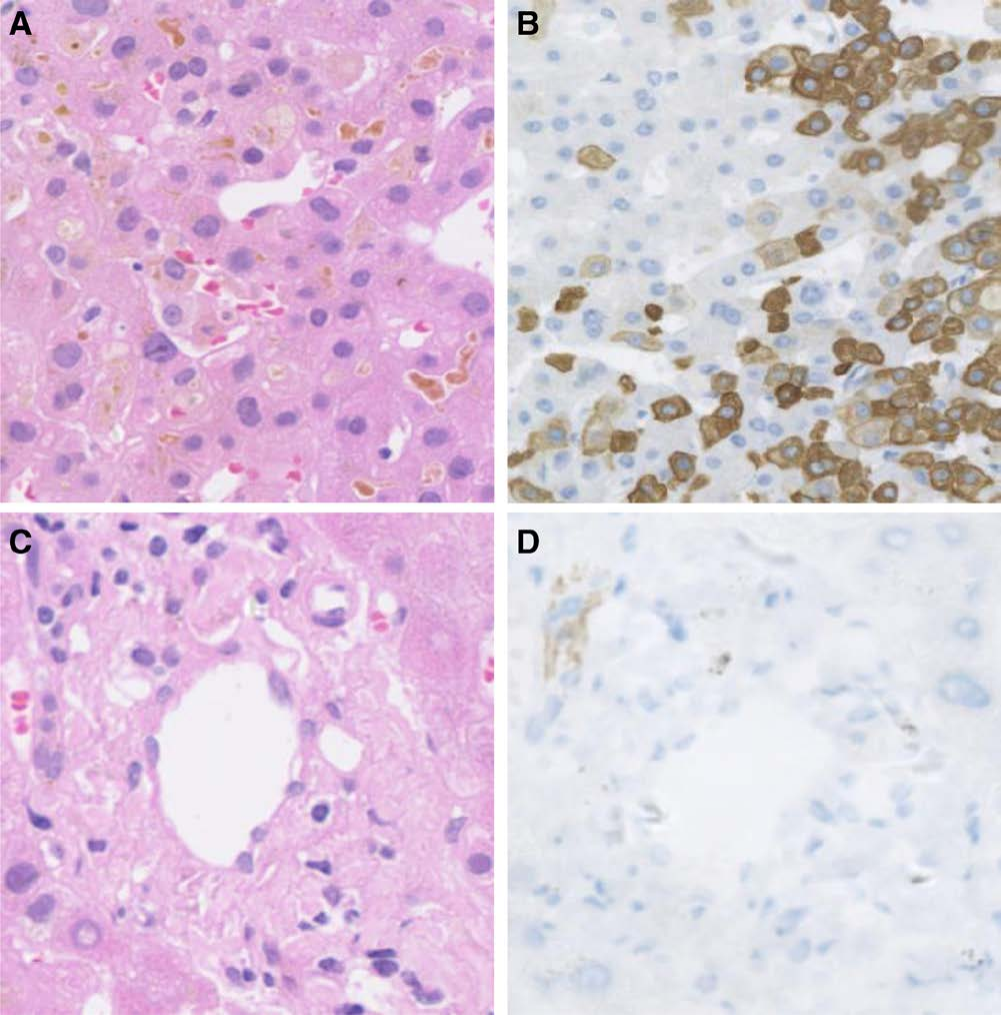
(A; HE × 200) A large number of hepatocytes showed cholestasis, with highly dilated capillary bile ducts and numerous small bile plugs. (B; CK7 × 200) Loss of interlobular bile ducts was observed in the portal tracts. Immunohistochemical staining for cytokeratin 7 (CK7) showed diffuse positivity in hepatocytes with varying staining intensity, presenting as acinar zone 1 to 3 cord-like distribution and single-cell scattered distribution. (C; HE × 200) Loss of bile duct structures in the portal tracts was observed. (D; CK19 × 200) Immunohistochemical staining for CK19 showed the absence of interlobular bile ducts in the portal tracts, with negative staining in hepatocytes.

A 67-year-old female presented to our hepatology department on November 29, 2021, complaining of generalized fatigue and reduced appetite persisting for over 2 months, along with jaundiced urine for over a month. She had previously been admitted to a local hospital on October 15, 2021, where liver function tests (LFTs) indicated markedly elevated bilirubin levels. Magnetic resonance imaging findings suggested hepatitis, gallstones, and gallbladder inflammation. The patient’s initial treatment regimen at the local hospital comprised magnesium isoglycyrrhizinate (150 mg daily) and reduced glutathione (2.4 g daily) for hepatoprotection, alongside ursodeoxycholic acid (UDCA, 250 mg b.i.d.) for cholestasis. Additionally, a 1-week course of prednisone (8 mg t.i.d.) was administered as anti-inflammatory therapy. Despite treatment, her symptoms persisted, and hyperbilirubinemia continued to worsen; the detailed trend in LFTs during prehospital is presented in Figure 1. She was subsequently transferred to our hospital for further management. Upon detailed inquiry, the patient reported a 10-year history of diabetes and had used a herbal decoction containing PF to relieve bone pain in September 2021. The dosage was 6 g per administration, twice daily, for a consecutive 1 week. There was no documented history of chronic liver disease, and previous routine LFTs had been within normal limits. Upon admission, physical examination revealed moderate jaundice of the skin and sclera, with no other notable findings. Considering her clinical history and presentation, a working diagnosis of drug-induced liver injury (mixed type, *R*-value 3.91, severity level 3) was made. To clarify the cause, several tests were conducted after admission. Viral hepatitis serology (hepatitis A, B, C, E) was negative. Epstein-Barr virus deoxyribonucleic acid and Cytomegalovirus deoxyribonucleic acid levels were within normal limits. Metabolic and tumor markers, autoantibody markers including anti-mitochondrial antibodies M2 type, anti-smooth muscle antibody, anti-liver-kidney microsome antibody, anti-soluble liver antigen antibody, and anti-glycoprotein-210 were negative. Abdominal computed tomography showed no significant intrahepatic or extrahepatic bile duct dilatation. Coagulation parameters were within normal range. A liver biopsy was performed to assess the underlying pathology. Histological analysis identified 16 portal areas, with small bile duct injury or interlobular bile duct loss observed in 10 of them; the portal areas showed prominent cholestasis, bile duct hyperplasia with marked dilatation, multiple micro-bile thrombi, and areas of focal multilobular necrosis. Immunohistochemical staining for cytokeratin 7 and CK19 revealed cholangiocytic metaplasia of numerous hepatocytes (70%), with loss of the majority of interlobular bile ducts. There was no evidence of steatosis or ballooning. Combined light and electron microscopy findings were consistent with cholestatic liver disease, most likely drug-induced (see Fig. 2).

**Figure 3.**
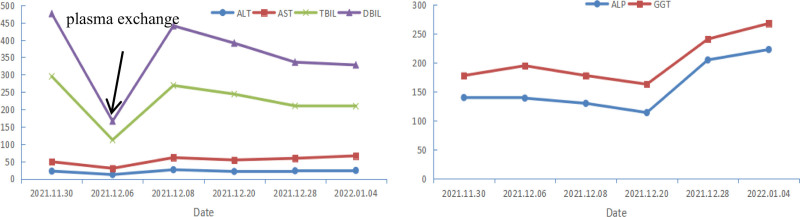
Trend of LFTs during admission. ALP = alkaline phosphatase, ALT= alanine aminotransferase, AST= aspartate aminotransferse, DBIL = direct bilirubin, GGT = gamma-glutamyl transferase, LFT = liver function test, TBIL = total bilirubin.

After admission, the patient received combination therapy for liver injury. The hepatoprotective regimen consisted of reduced glutathione (2.4 g daily) and magnesium isoglycyrrhizinate (200 mg daily). For cholestasis, the patient was prescribed ademetionine 1,4-butanedisulfonate (1000 mg daily) and UDCA (250 mg t.i.d). However, the jaundice failed to improve significantly. On December 8, 2021, therapeutic plasma exchange was performed with 2500 mL of fresh frozen plasma. This resulted in only a transient reduction in bilirubin levels, which rebounded shortly thereafter. Considering that the patient’s current condition is dominated by cholestasis, it was decided to streamline the medications. On December 15, 2021, her medication was changed to alprostadil (10 µg daily) to improve hepatic microcirculation, while UDCA (250 mg t.i.d.) was continued. The patient showed improvement in appetite, nausea, and pruritus, along with better overall mental status. Follow-up LFTs revealed a gradual reduction in bilirubin levels, and the detailed trend in LFTs during admission is presented in Figure 3.

Before discharge, LFTs showed an albumin level of 30.2 g/L, total bilirubin at 151.2 µmol/L, direct bilirubin at 119.7 µmol/L, alkaline phosphatase (ALP) at 205 U/L, and gamma-glutamyl transferase (GGT) at 45 U/L. The patient was discharged on January 4, 2022, with a continued regimen of glutathione for hepatoprotection, alongside ursodeoxycholic acid (250 mg bid). Post-discharge monitoring was maintained. On April 9, 2022, LFTs indicated normalized levels of bilirubin and aminotransferases, although ALP and GGT were elevated at 436 and 497 U/L, respectively. By October 24, 2024, bilirubin and aminotransferase levels remained within the normal range, with ALP at 251 U/L and GGT at 173 U/L. The trend of LFTs during the follow-up period is presented in Table 1.

**Table 1:** Trend of LFTs during the follow-up period.

Follow-up period timepoint	TBIL (μmol/L)	DBIL (μmol/L)	ALT (U/L)	AST (U/L)	ALP (U/L)	GGT (U/L)
At Discharge (January 4, 2021)	143	119	42	24	223	45
Post-discharge follow-up						
April 9, 2022	27	14	84	65	436	497
October 24, 2024	26	8	66	41	251	173

ALP = alkaline phosphatase, ALT= alanine aminotransferase, AST= aspartate aminotransferse, DBIL = direct bilirubin, GGT = gamma-glutamyl transferase, LFT = liver function test, TBIL = total bilirubin.

## 3. Discussion

A study found that PF could speed up fracture healing by promoting osteoclast bone resorption and osteoblast bone diferentiation through the extracellular signal-regulated kinase pathway^[7]^; there have been an increasing number of reports of liver damage in recent years with the increase of PF’s clinical use,^[8–13]^ and the clinical features seen in these patients are listed in Table 2. Based on the analysis of these studies, we found that PF-induced liver injury predominantly manifests as cholestatic liver injury. Most cases tie liver injury to prolonged use (over 2 months) or high doses (above 15 g/d); discontinuing PF early and using cholestasis-targeted therapies usually leads to good recovery. However, PF-induced VBDS is rare, as seen in our case, PF-induced liver injury involves many mechanisms, such as oxidative damage, cholestasis, mitochondrial dysfunction, metabolic disorders, and aberrant liver regeneration.^[6]^ The drug can induce bile duct reduction through several postulated mechanisms, including: direct assault on bile duct cells by drugs or their toxic metabolites upon release into bile; continuous exposure to toxic bile salts when the protective defense barrier of bile duct epithelium is damaged; and drug-induced hypersensitivity of T lymphocytes to attack bile duct epithelial cells.^[14,15]^ Pathologically, histological diagnosis of ductopenia should be performed when the pathologist documents absence of interlobular bile ducts in at least 50% of 10 or more well-formed portal tracts. The extent of bile duct disappearance in D-VBDS can exhibit stability or progression, with certain cases demonstrating distinctive features of chronic gallbladder stasis within the hepatic parenchyma, as manifested by bile duct disappearance, such as gallbladder stasis with chrysanthemum accumulation and bile salt deposition with hepatocytes around the portal area.^[16,17]^ The prognosis of VBDS mainly depends on the etiology. Numerous studies^[3,5,18–20]^ have confirmed that the prognosis of D-VBDS is closely related to the extent of bile duct disappearance and the level of bile duct injury. Patients with extensive damage and significant bile duct injury are associated with a poor prognosis, with severe cases potentially progressing to acute liver failure requiring liver transplantation.^[21–23]^ Notably, due to the regenerative capacity and reparative abilities of small bile ducts, the prognosis of patients with small bile duct injury and terminal bile duct injury is satisfactory.

**Table 2:** Comparison of cases of liver injury caused by Psoraleae Fructus in the literature.

Author (yr)	Age (yr)/sex	Dosage	Duration	Chief features	Liver function normalization time (d)	Prognosis
Nam SW et al 2005^[8]^	44/F	A large dose	7 wk	Nausea, vomiting, and general weakness for 1 mo.	133	Good
Cheung WI et al 2009 (Case 1)^[9]^	39/M	Normal	2 mo	Jaundice,anorexia, and abdominal discomfort for 1 wk	35	Better
Cheung WI et al 2009 (Case 2)^[9]^	22/M	Normal	2 mo	Jaundice and tea-colored urine for 3 d	63	Good
Cheung WI et al 2009 (Case 3)^[9]^	20/F	Normal	2 wk	Fever, epigastric pain, and jaundice for 1 wk, and palpable liver edge on examination	28	Better
Teschke R et al 2009^[10]^	20/F	Normal	9 mo	Jaundice in the sclera	70	Good
Smith DA et al 2014^[11]^	52/F	/	/	Jaundice, vomiting, pruritus, and abdominal pain for 1 wk	90	Good
Li et al 2019^[12]^	53/F	Normal	7 mo	Weakness, nausea, and vomiting for 3 d; severe diffuse skin and scleral yellowing	Died 5 days after admission	Poor (death)
Rong JC et al 2020 (12 cases in cohort)^[13]^	54–77/9F, 3M	/	Median 30 d	Jaundice (all cases); accompanied by fatigue (7cases), nausea/vomiting (6 cases), abdominal pain (5 cases), and hepatic encephalopathy (2 cases)	8 cases recovered, median 61 d	12 cases: 8 good, 2 better, 2 poor (death)

The patient, a middle-aged and elderly female, presented clinical symptoms including weakness, decreased appetite, and pruritus. Liver function tests showed elevated bilirubin, mainly direct bilirubin. Oral Chinese herbs were taken to treat bone pain within 2 months before the onset of the disease, with no evidence for extrahepatic biliary obstructive diseases. Considering the high possibility of drug-induced liver damage, further refinement of liver biopsy through liver puncture was deemed necessary. Microscopic examination of the pathological findings revealed bile duct disappearance and cholestatic alterations, which can also be observed in the histopathology of common cholestatic liver conditions such as primary biliary cholangitis (PBC). However, the patient did not exhibit the characteristic pathology of PBC, such as peri-biliary granuloma.^[24]^ Moreover, anti-mitochondrial antibodies M2 type, anti-glycoprotein-210, and other antibodies were negative, suggesting insufficient evidence for a diagnosis of PBC. In contrast, congenital cholestasis diseases such as progressive familial intrahepatic cholestasis and Dubin-Johnson typically manifest at a younger age, lacking features of bile duct loss and hepatocyte injury on liver histopathology. These conditions are characterized by elevated direct bilirubin levels without abnormalities in other liver function markers and the absence of skin pruritus. Therefore, the possibility of congenital intrahepatic cholestasis was deemed low.^[25]^ The comprehensive analysis considered that the patient was highly likely to have drug-related liver function impairment, and the RACUM score was 5 points, indicating a likelihood of drug-related liver injury. The calculated *R*-value of 3.94 signified that the initial liver dysfunction in the patient primarily involved hepatocyte damage, progressively evolving into cholestatic features such as elevated direct bilirubin, bile acid levels, and skin pruritus.

The main treatment of D-VBDS involves discontinuation of suspected causative medications. Currently, there are no specific drugs to induce bile duct regeneration, and thus, management strategies mainly focus on liver protection, reduction of enzyme levels, enhancement of gallbladder function, immunosuppressive therapy, and implementation of artificial liver support when necessary. Immune dysregulation is widely acknowledged as a key pathogenic mechanism in D-VBDS.^[2,26]^ Therefore, immunosuppressants have significant effects on VBDS caused by multiple causes, particularly in cases where autoimmunity is involved. Glucocorticoids are the preferred drugs, with mycophenolate mofetil considered as an alternative if glucocorticoids prove ineffective. The patient used hormones when seeking medical treatment in other hospitals, but the regimen was non-standardized and yielded suboptimal results. Notably, the patient tested negative for autoimmune-related antibodies, exhibited normal serum globulin levels, and liver histopathology did not reveal features of spontaneous liver diseases characterized by interface inflammation with prominent lymphocytic and plasma cell infiltration. Furthermore, considering the patient’s existing diabetes condition, the use of hormones could complicate blood sugar control. Consequently, hormone therapy was not included in the patient’s subsequent treatment at our hospital. Meanwhile, analysis of previous case reports on *Psoralea corylifolia*-related liver injury shows that none of these cases received hormone therapy either.

The cholestasis indicators such as ALP and GGT increased significantly at the initial examination of the patient’s onset. However, as the disease progressed, the patient’s bilirubin levels gradually increased, while ALP and GGT gradually decreased. This trend could potentially be associated with the prior use of glucocorticoids and choleretic medications administered at other medical facilities during the onset of the patient’s illness. Meanwhile, relevant studies suggested that some patients with cholestatic liver disease had severe bile stasis in the later stage. Total bilirubin and TBA gradually increased, while ALP and GGT levels showed a declining trend. The decreased ALP and GGT levels did not mean the recovery of bile duct injury but indicated the aggravation of the lesions.^[15,27]^ Subsequent liver function evaluations post-discharge revealed a gradual reduction in jaundice to within normal limits. However, ALP and GGT levels gradually increased again, indicating that the small bile duct of the patient had significantly regenerated and the function of the small bile duct had recovered. Nevertheless, the regenerated small bile duct was insufficient to fully alleviate cholestasis. The patient subsequently developed chronic cholestasis, a common manifestation of chronic drug-induced liver damage. Over time, there remains a risk of progression to cirrhosis, underscoring the necessity for ongoing regular follow-up assessments. If warranted, a repeat liver puncture biopsy should be considered for further review.

## Acknowledgments

We would like to thank Qin Jing from the Department of Pathology, Changde No.1 People’s Hospital, for her assistance.

## Author contributions

**Conceptualization:** Qing-Hai Wang, Xi-Yang Dong.

**Investigation:** Chuang Lei.

**Methodology:** Min Liu.

**Project administration:** Xi-Yang Dong.

**Resources:** Hong-Ling Tian.

**Software:** Chuang Lei.

**Validation:** Hong-Ling Tian.

**Writing – original draft:** Min Liu.

**Writing – review & editing:** Xi-Yang Dong.
